# PRAGMATIC IMPLEMENTATION OF AN ADVANCE CARE PLANNING INITIATIVE: MOVING KNOWLEDGE INTO ACTION

**DOI:** 10.1093/geroni/igad104.1528

**Published:** 2023-12-21

**Authors:** Kelly Smith, Jessica Colburn, Daniel Scerpella, Maura McGuire, Kathryn Walker, Jennifer Wolff

**Affiliations:** University of Toronto, Toronto, Ontario, Canada; Johns Hopkins, Baltimore, Maryland, United States; Johns Hopkins Bloomberg School of Public Health, Baltimore, Maryland, United States; Johns Hopkins Community Physicians, Baltimore, Maryland, United States; MedStar Health, Columbia, Maryland, United States; Johns Hopkins University, Baltimore, Maryland, United States

## Abstract

Primary care is an important setting for patient and family-centered communication with older adults given the high frequency of interactions, longitudinal trusted relationships, and patient preferences for advance care planning (ACP). Increasing ACP conversations and documentation of advance directives in primary care is a focus of quality-of-care initiatives and is particularly relevant for older adults, including those with dementia. The SHARING Choices ACP program evaluation was designed to examine the fidelity of implementation with the two partner health systems. SHARING Choices includes a mailing (introductory letter, an agenda-setting checklist, a blank advance directive, and information about shared-portal access for family) sent two weeks prior to scheduled primary care visits for patients aged 65 and above along with access to an ACP facilitator in each practice. Embedding SHARING Choices within primary care practices involved a multi-step process, including onboarding of health system partners, facilitator identification and training, tailored onboarding, practice champion engagement, and practice orientation and ongoing support. Of 23,220 candidate patients, 17,931 outreach attempts by phone (77.9%) and the patient portal (22.1%) were made by ACP facilitators and 645 conversations occurred. Most conversations (94.8%) were less than 45 minutes in duration. Study findings reinforce the value of adaptable study design; co-designing workflow adaptations with frontline staff; adapting implementation processes to fit the operational and organizational priorities of the health system.

